# Discriminant analysis as a tool to classify farm hay in dairy farms

**DOI:** 10.1371/journal.pone.0294468

**Published:** 2023-11-28

**Authors:** Aldo Dal Prà, Riccardo Bozzi, Silvia Parrini, Alessandra Immovilli, Roberto Davolio, Fabrizio Ruozzi, Maria Chiara Fabbri

**Affiliations:** 1 Centro Ricerche Produzioni Animali—Soc. Cons. p. A., Reggio Emilia, Italy; 2 Institute of BioEconomy-National Research Council (IBE-CNR), Florence, Italy; 3 Department of Agriculture, Food, Environment and Forestry (DAGRI), University of Florence, Florence, Italy; 4 Fondazione CRPA Studi Ricerche—ETS, Reggio Emilia, Italy; University of Agriculture Faisalabad, PAKISTAN

## Abstract

Hay is one of the primary constituents of ruminant feed, and rapid classification systems of nutritional value are essential. A reliable approach to evaluating hay quality is a combination of visual combined inspection by NIRS analysis. The analysis was carried out on 1,639 samples of hay collected from 2016 to 2021 in northern Italy. Discriminant analysis (DAPC) on five hay types (FOM, forage mixtures; APG, first alfalfa cutting with prevalence of graminaceous >50%; PRA, prevailing alfalfa >50%; PUA, purity alfalfa >95%; and PEM, permanent meadows) was performed by *ex-ante* visual inspection categorization and NIRS analysis. This study aimed to provide a complementary method to differentiate hay types and classify unknown samples. Two scenarios were used: i) all data were used for model training, and the discriminant functions were extracted based on all samples; ii) the assignment of each group was assessed without samples belonging to the training set group. DAPC model resulted in an overall assignment success rate of 66%; precisely, the success was 84, 79, 69, 37, and 27% for PUA, FOM, PRA, APG, and PEM, respectively. In the second scenario, three groups showed percentages of posterior assignment probability higher than 70% to only one group: PUA with PRA (~ 99%), PRA with PUA (~71%), and PEM with FOM (~75%). Discriminant analysis can be successfully used to differentiate hay types and could also be used to assess factors related to hay quality in addition to NIRS analysis.

## 1. Introduction

More than 50% of dairy cows producing more than 70% of Italy’s milk are bred in the Po Valley of Northern Italy, where the Parmigiano Reggiano PDO cheese (PR) production area is located. In 2022, about 4.002 million wheels of PR were produced, using more than 18% of the whole milk produced in Italy [1].

The study areas, located on the south side of the Po River, is mainly characterized by alfalfa (*Medicago sativa* L.) managed without the use of herbicides and representing the prevailing crop of the dairy farms, Italian ryegrass (mainly *Lolium multiflorum* Lam.), forage mixtures (mainly with *Lolium* spp. and winter cereals), and permanent meadows forage cultivations (that are still found in the less intensive farms) [2]. These crops produce hay to prevent *Clostridia* contamination and potential swelling defects in the preservative-free cheese subjected to length seasoning [3]. The importance of hay in the production system of PR is therefore significant; forages are highly variable in chemical composition, digestibility, and potential intake [4, 5], although the Italian hays are often of low quality and reduced nutrient value due to climatic conditions [6]. A feeding strategy that requires a forage: concentrate ratio equal to 70 : 30, with use of silages prohibited, has also recently been introduced in Italy [7]. High quality hay in ruminant ration can improve the rumen fermentation characteristics, inflammatory state, and oxidative stress, positively effecting animal well-being [8, 9].

Feed nutritive quality is associated with nutrient contents, nutrient digestibility, and the amount of forage consumed [10, 11], and these effects are strongly correlated with the stage of maturity and botanical composition [10, 12–14]. Considering that the taxonomic group and the phenological state affect rumen digests of nutrients, the most reliable way to evaluate hay for quality could be a combination of physical-sensory inspection and chemical analysis.

A broad description of hays used for in situ analysis was made by Hackmann et al. [15], where hays were also divided for cutting. An accurate and rapid estimation of chemical composition is critical to formulate diets that meet animal requirements [5, 16] and forage quality evaluation based on dynamic nutritional models [17, 18] or on net energy for lactation (NE_L_) [19, 20]. Also, biological parameters, such as *in situ* rumen NDF degradation characteristics, have been proposed to properly evaluate forages used in ruminants [21–23]. In this context, Near Infrared Reflectance Spectroscopy (NIRS) has been used in several agronomic applications for both quantitative and qualitative analyses [24]; in particular, forage classification has been developed by Gallo et al. [25]. A principal component analysis can be helpful for identifying which variables have more weight in the definition of hay/forage quality and identifying the percentage of successful assignments considering the numerous parameters that could affect the results. The combination of visual inspection with NIR analysis is a reliable approach that could be useful both to formulate the ruminant diet and pay the right price at the time of farmers ‘purchase.

The visual determination of the botanical composition of a fodder crop is a time-consuming operation that has an inherent approximation due to the subjectivity of the evaluator. This assumption also applies when the assessment is carried out to identify the prevalence of some families.

To date, the technology allows to implement NIRS systems also on agricultural equipment operating in the open field. The instantaneous evaluation of hay in terms of composition, and consequently of nutritional value, can be decisive both in the subsequent use of hay to compose the ration and to define a market value of the product itself.

Indeed, the objective of this study was to evaluate a rapid classification system of the nutritional value and quality of hay, as well as their prevalent functional group.

## 2. Materials and methods

### 2.1 Study areas

**Fig 1:**
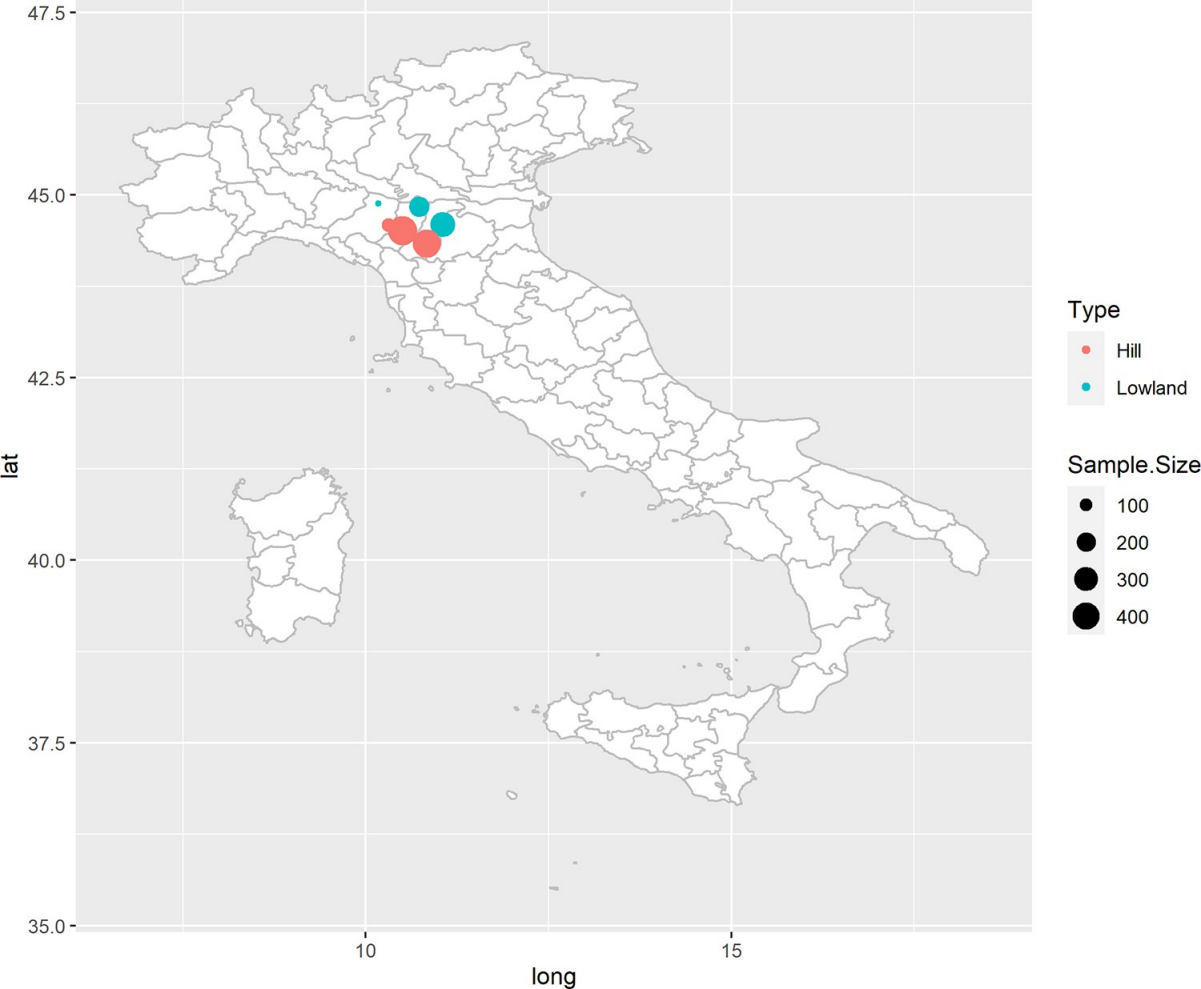
Hay sampling sites showing the number of samples.

Emilia-Romagna is located in Northern Italy, South of the Po river valley, and in the plain area its land use is mostly dedicated to intensive agriculture with strong connections with the agro-food sector. Apennines mountains are present in the southern part of the region, which contribute to the regional production by means of low input agriculture, pasture and forest areas. The samples were taken in the provinces of Modena, Parma, and Reggio Emilia (Fig 1). Temperature and rainfall data were obtained from the regional meteorological agencies [26] at a site representative (10°51’06.23’ N 44°68’95.29’ E, altitude 95 m a.s.l.) of the wider study area, recording an annual mean temperature of 14.6°C in 2016, 15.0°C in 2017, 15.3 in 2018, 13.5 in 2019, 12.8 in 2020, and 13.6°C in 2021, and annual total rainfall of ~689 mm, ~560 mm, ~1.020 mm, ~1.038 mm, ~850 mm, and ~232 mm from 2016 to 2021 (S1 Fig). The soils have a remarkable variability above all between the plain areas and those of hills [27].

### 2.2 Sampling procedure and visual inspection

A set of 1.639 dried hays were randomly collected from different locations in the Parmigiano Reggiano area from the 2016 to 2021 harvest seasons. The hay samples were analyzed at the CRPA Lab, Reggio Emilia, Italy. Samples were taken with a cylindrical probe, and at least three probes were pooled in a single cotton bag, mixed thoroughly, and finally, sent to a laboratory within 24 h for analysis. The manual sorting method was designed in the lab and consisted of visual inspection and sensory-physical analysis to classify hays before the examination. Dried hays were spread on a tray with a white base, and the classification was assigned based on the five types below described, based on the percentage of the prevalent species. The hay samples were classified into:

- purity alfalfa >95% (PUA), n = 421;
- prevailing alfalfa >50% (PRA), n = 349;
- first alfalfa cutting with a prevalence of graminaceous >50% (APG), n = 275;
- permanent meadows (PEM), n = 172;
- forage mixtures (FOM), n = 422.

### 2.3 NIRS analysis

To estimate the nutritional value, hay samples were ground to 2 mm, and the spectra acquisition was performed using the instrument Foss NIR-System 5000 monochromator (NIR-System, Silver Spring, MD, USA) using the spinning ring cup cell as sample as sample transport module by double scanning each sample in the 1098–2500 nm spectral region. Mathematical treatments of spectral data were processed using WinISI II V1.5 software (Infrasoft International, Port Matilda, PA, USA). NIRS calibration equations were developed for the predict in vitro NDF parameters of Italian hay. Predictions were performed using the equation developed and validated by Brogna et al. [28] and Palmonari et al. [6], considering a coefficient of determination higher than 0.85 and a standard error ranging from 1.9 and 3.4 in cross-validation. The NIRS curve (.cal editable file) is periodically validated with chemical analyses based on official methods and in vivo NDF parameters. Indeed, the following parameters were recorded: dry matter (DM, %), ash (% of DM), crude protein content (% of DM), neutral detergent-insoluble protein (% of DM), acid detergent-insoluble protein (% of DM), and soluble protein (% of DM) according by [29, 30], neutral detergent fiber with amylase and sodium sulfite method, according to Mertens [31], (NDF, % of DM), acid detergent fiber (ADF, % of DM), acid detergent lignin (ADL, % of DM), undigested NDF after 240 h (uNDF, % of aNDFom, as reported by [28], dNDF digestible NDF evaluated after 24 h of in situ rumen incubations, as reported by [32], fat (% of DM), starch (% of DM), sugar (% of DM), metals and other elements (Ca, P, Mg, and K) as recommended by [33], and net energy for lactation (NE_L_, kcal kg DM^−1^).

### 2.4 Principal components analysis and discriminant analysis of principal components

Missing and abnormal values were discarded from the dataset. A correlation plot was elaborated as preliminary control to verify the correct application of a multivariate method like discriminant analysis [34]. After that, a Principal Components Analysis (PCA) was conducted using the *prcomp* function of the R package [35] on the 19 parameters estimated. Discriminant analysis was chosen as approach because it allows the membership identification of observations to a group of origin, independently to potential factors of noise (e.g. climate condition, soil composition, regions of sampling). The aim was to assess the group identification of the hay.

To achieve this, the methodology of discriminant analysis of principal components (DAPC) implemented in the *adegenet* R package was adopted [36]. DAPC comprises two steps: firstly, a PCA is conducted, and then, a small number of selected PCs (instead of the original physical-sensorial traits) is used as input for the linear discriminant analysis (LDA). The selection of the optimal number of PCs to be further used in the LDA is made via cross-validation (CV), where the data is split into training (TRN), and validation (VAL) sets (70 and 30% respectively in this study). The following criteria were implemented for the selection of PCs: i) the number of replicates to be carried out at each level of PC retention was set to 30, ii) a maximum number of 19PCs were tested, iii) the number of PCs to be retained was based on number of PCs associated with the highest mean success. Two different scenarios of DAPC were applied. Scenario 1: the whole dataset was analyzed simultaneously; in this scenario, all available data were used for model training, and the discriminant functions were extracted based on all samples. Scenario 2: assignment of each group without the presence of any samples belonging to the group tested in the training set (external validation).

## 3. Results

### 3.1 Visual inspection

A set of 1,639 hay samples identified with the visual classification were thus clustered: 422 FOM, forage mixtures, 275 APG, first alfalfa cutting with a prevalence of graminaceous>50%, 349 PRA, prevailing alfalfa >50%, 421 PUA, purity alfalfa >95%, and 172 PEM, permanent meadows.

### 3.2 NIRS analysis

The descriptive statistics data (mean and SD), as predicted by NIRS, is shown in Table 1.

**Table 1:** Descriptive analysis of hay data.

Item*	FOM	APG	PRA	PUA	PEM
	Mean—*s*.*d*.				
DM (DM, %)	89.96–9.52	91.38–1.37	88.83–11.28	89.48–5.75	90.10–5.99
Ash (% of DM)	9.48–1.85	9.49–1.27	10.27–1.78	10.43–1.39	8.97–1.37
CP (% of DM)	9.41–2.30	11.95–3.11	16.67–1.74	18.96–1.87	9.39–2.11
NDIP (% of DM)	2.61–0.84	2.84–0.87	3.23–0.95	3.51–1.04	2.84–0.83
ADIP (% of DM)	1.34–0.24	1.49–0.26	1.60–0.21	1.65–0.22	1.39–0.22
SolP (% of DM)	3.73–1.27	4.34–1.33	6.33–1.08	7.00–1.26	3.20–1.02
aNDFom (% of DM)	58.16–5.32	53.49–6.05	45.77–4.71	42.89–4.72	57.63–4.98
ADF (% of DM)	40.16–3.83	39.61–3.93	38.12–3.71	36.56–3.73	39.96–3.98
ADL (% of DM)	5.91–1.11	6.74–1.40	7.83–1.02	7.89–0.96	6.21–1.07
uNDF (% of aNDFom)	30.75–8.46	36.44–11.00	50.41–9.15	51.50–7.61	28.30–7.19
dNDF (% of aNDFom)	65.50–6.27	58.93–9.71	46.46–7.68	44.18–6.34	65.24–6.26
Fat (% of DM)	1.45–0.34	1.52–0.32	1.64–0.31	1.60–0.28	1.51–0.34
Starch (% of DM)	1.78–1.74	1.52–0.51	1.37–0.55	1.54–0.57	1.57–0.49
Sugar (% of DM)	8.40–2.52	7.77–2.35	6.24–1.72	6.64–1.71	8.60–2.35
Ca (% of DM)	0.67–0.25	0.91–0.33	1.43–0.23	1.53–0.21	0.65–0.23
P (% of DM)	0.28–0.06	0.29–0.05	0.28–0.04	0.31–0.04	0.28–0.07
Mg (% of DM)	0.15–0.05	0.17–0.04	0.22–0.04	0.24–0.04	0.19–0.07
K (% of DM)	2.32–0.63	2.40–0.50	2.52–0.44	2.60–0.47	2.16–0.61
NEL (kcal kg DM^-1^)	1106–183	1119–208	1167–213	1199–239	1131–98

Forage mixtures (FOM); First alfalfa cutting with the prevalence of graminaceous >50% (APG); Prevailing alfalfa >50% (PRA); Purity alfalfa >95% (PUA); Permanent meadows (PEM).

*DM, dry matter, CP, crude protein content, NDIP, neutral detergent-insoluble protein, ADIP, acid detergent-insoluble protein, SolP, soluble protein, aNDFom, neutral detergent fiber with α-amylase, sodium sulfite, and correcting for ash contamination, ADF, acid detergent fiber, ADL, acid detergent lignin, uNDF, undigested NDF after 240h of in situ rumen incubations, dNDF, digestible NDF evaluated after 24h of in situ rumen incubations, NE_L_, net energy for lactation.

Hay groups characterized by alfalfa (PRA and PUA) showed high protein contents and lower levels of aNDFom with lower digestibility after 24h of in situ rumen incubations (ADL negatively affects forage fiber digestion). Conversely, FOM, APG, and PEM showed high aNDFom digestibility and lower uNDF content. Grass hays (FOM and PEM) presented lower calcium, magnesium, and potassium, whereas higher PRA and PUA contents.

### 3.3 Principal components analysis and Discriminant analysis of principal components

**Fig 2:**
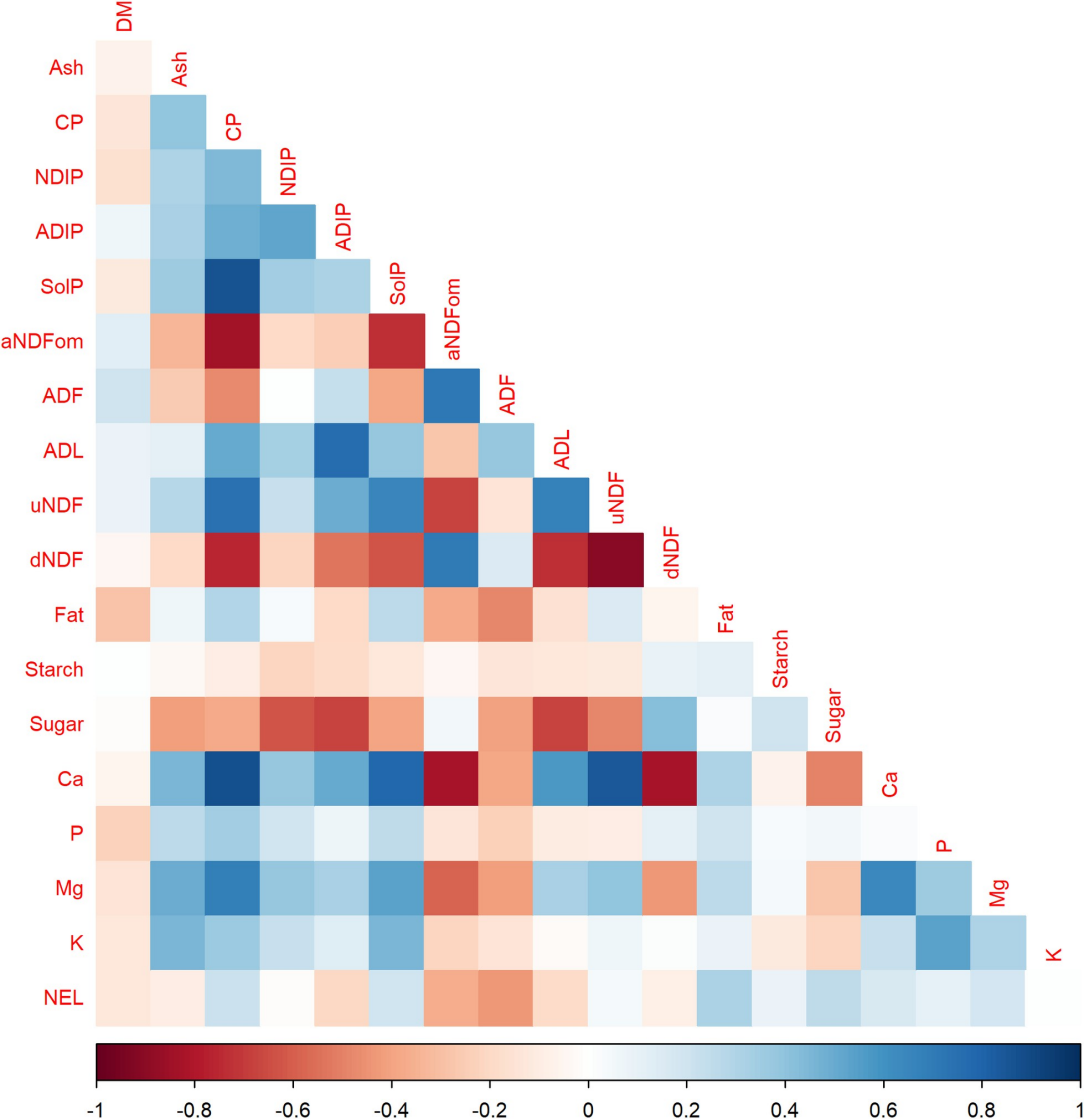
Correlation plot of all parameters analyzed, where: DM, dry matter, CP, crude protein content, NDIP, neutral detergent-insoluble protein, ADIP, acid detergent-insoluble protein, SolP, soluble protein, aNDFom, neutral detergent fiber with α-amylase, sodium sulfite, and correcting for ash contamination, ADF, acid detergent fiber, ADL, acid detergent lignin, uNDF, undigested NDF after 240h of in situ rumen incubations, dNDF, digestible NDF evaluated after 24h of in situ rumen incubations, NE_L_, net energy for lactation.

After quality control of data, 1,589 samples remained for further analysis. Fig 2 shows the correlation between traits. A negative correlation between hay uNDF and dNDF indicates that uNDF is a useful parameter for the prediction of the nutritive value of hay; uNDF is also negatively correlated with sugar. The dNDF value is negatively correlated with CP, ADL, and SolP. In composing grass-alfalfa mixtures (PRA, PEM, and FOM), the characteristics of plant species and their nutritional value are very different. However, some essential correlations, such as the digestibility of forages with NDF and its lignification, are still observed. Furthermore, minerals Mg and Ca were negatively correlated with dNDF and aNDFom parameters.

**Fig 3:**
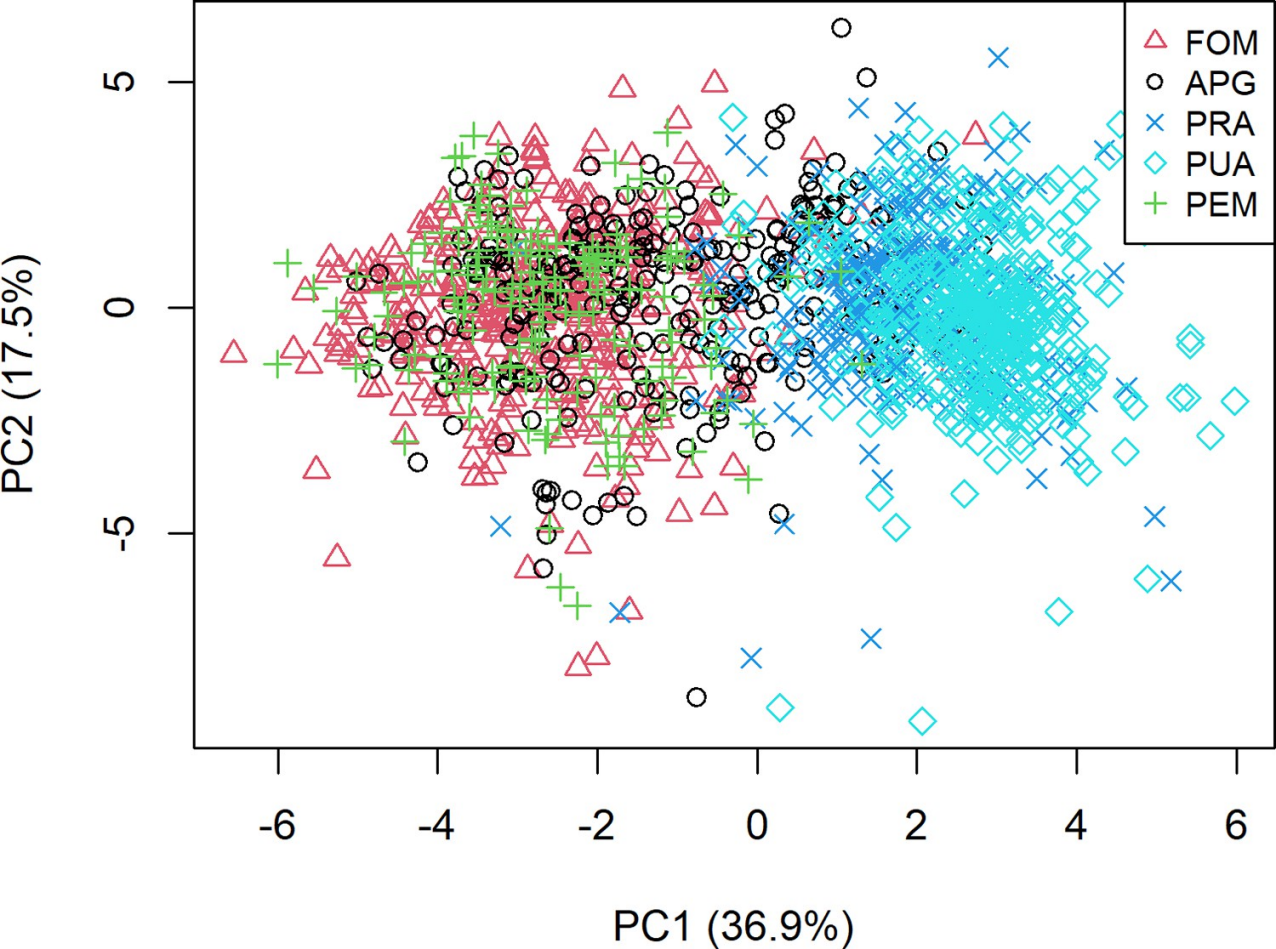
Biplot of principal components analysis, where FOM is forage mixtures, APG is first alfalfa cutting with the prevalence of graminaceous >50%, PRA is prevailing alfalfa >50%, PUA is purity alfalfa >95%, PEM is permanent meadows.

Regarding PCA, two components were retained, explaining 54.4% of the total variability of the dataset. Fig 3 reports the two-dimensional plot characterized each cluster for their position on PC1 and PC2. The PC1 allowed the identification of the two main populations: PUA and PRA opposed to FOM, APG, and PEM. Fig 2 identifies the transition of PRA from PUA to other groups with ryegrass. Otherwise, PC2 was not able to separate the five groups analyzed. PUA, shown in Fig 2, is represented in a specific cluster. Grasses grow slower than alfalfa, reducing their proportion in subsequent cuttings. This condition was represented by the two PRA groups and the cluster that could define the evolution of the cultivation of alfalfa over time, with the increase of perennial meadows, APG. The PEM of permanent grasslands represented a well-defined subgroup of FOM. The FOM formed a group with high variability, and the origin of hay samples from commercial forage mixtures explained the result.

**Fig 4:**
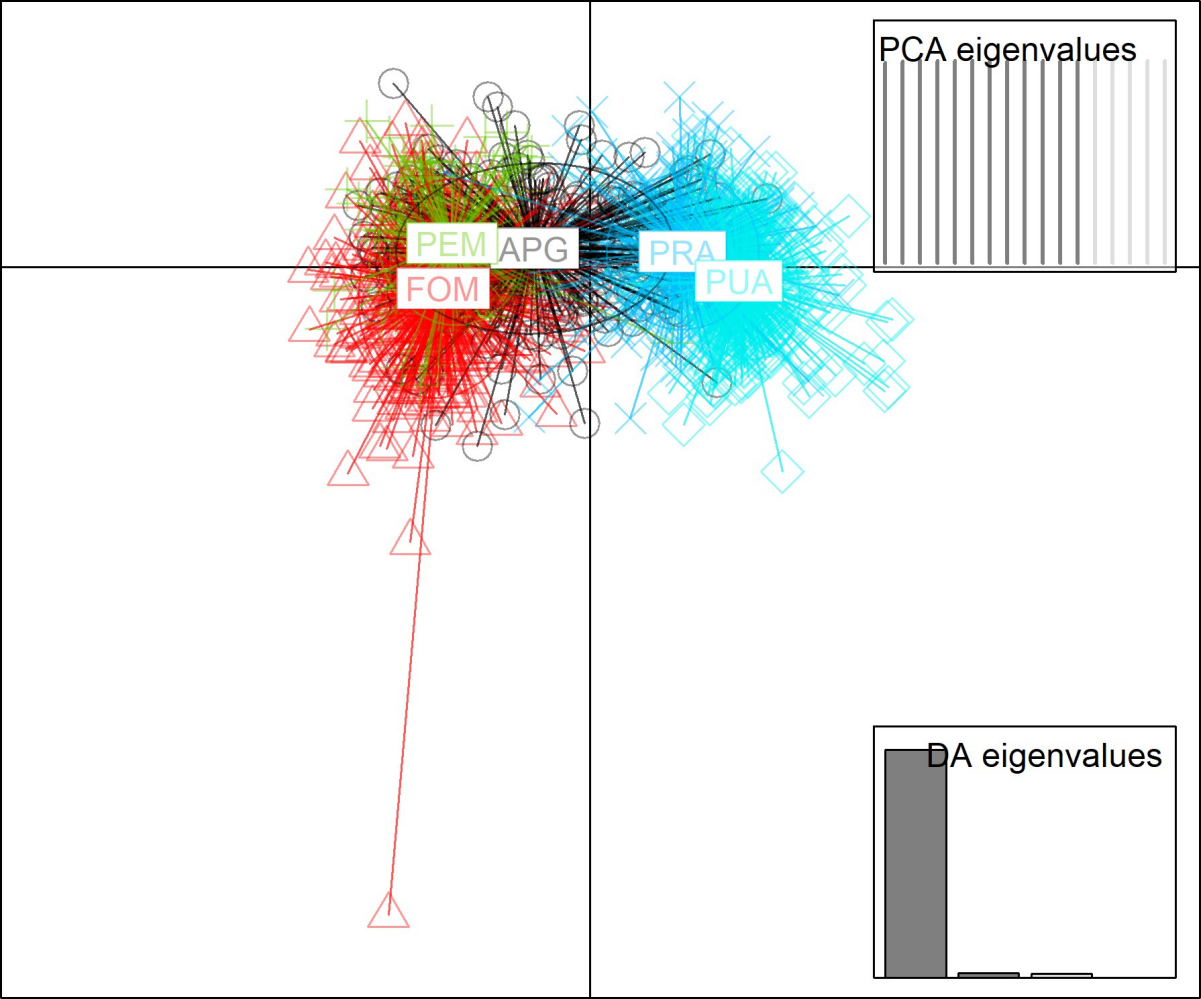
Biplot of DAPC analysis, where FOM is forage mixtures, APG is first alfalfa cutting with the prevalence of graminaceous >50%, PRA is prevailing alfalfa >50%, PUA is purity alfalfa >95%, PEM is permanent meadows.

In the first scenario of DAPC, all data were used. With this approach, it is possible to derive group membership probabilities, which can be interpreted to assess how clear-cut or admixed the clusters are. The pattern of the diversity based on DAPC is presented in Fig 4, where clusters have been better identified than PCA; PUA and PRA represented a separated cluster characterized by lower variability. APG shaped an intermediate cluster nearer to PEM and FOM.

The first 12 PCs, explaining ~60% of the total variability in the data, were used in the final DAPC model, resulting in an overall assignment success rate of 66% to the group of origin.

**Fig 5:**
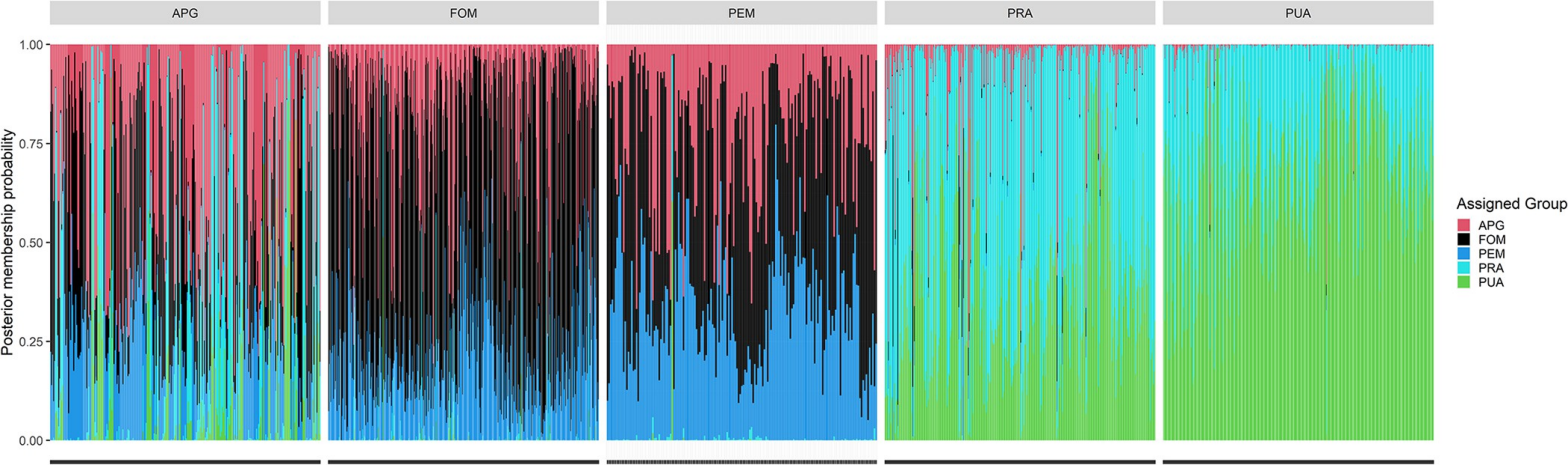
Posterior membership probability of each sample where FOM is forage mixtures, APG is first alfalfa cutting with the prevalence of graminaceous >50%, PRA is prevailing alfalfa >50%, PUA is purity alfalfa >95%, PEM is permanent meadows.

The assignment success for PUA was the highest (84%) among the groups, followed by FOM (79%), PRA (69%), APG (37%), and finally, PEM, with an overall assignment success rate of 27% to the group of origin. The assignment of each sample is presented in Fig 5, having a global picture of the clusters’ composition. Each barplot represents a sample, and the proportion of color height was the calculated average probability of assignment of that specific group.

The posterior assignment probability for each sample allowed to highlight the similarity among groups. The APG resulted in a very heterogeneous group, while for FOM and PEM, the overlapping was evident, underlining the difficulty distinguishing samples from these two highly admixed groups.

In Scenario 2, a variable number of PCs was used for each group. The number of training and validation test samples and the number of PCs used are reported in Table 2.

**Table 2:** Number of samples for training (TRN) and validation (VAL) sets and number of PCs used for each group.

Group ^a^	TRN set size	VAL set size	n. PCs
FOM	1201	388	10
APG	1320	269	12
PRA	1244	345	12
PUA	1169	420	12
PEM	1422	167	11

^**a**^Forage mixtures (FOM); First alfalfa cutting with prevalence of graminaceous >50% (APG); Prevailing alfalfa >50% (PRA); Purity alfalfa >95% (PUA); Permanent meadows (PEM).

The percentages of posterior assignment probability are presented in Table 3: it was paired because, in Scenario 2, the VAL set was not included in the TRN set. Three groups showed a percentage of assignment higher than 70% to only one group: PUA with PRA (~ 99%), PRA with PUA (~71%), and PEM with FOM (~75%). Also, FOM was assigned 65% of the time to PEM and the remaining 33% to APG. This latter group had the lowest percentage of assignments with PUA. As expected, in many instances, there was no reciprocity in the pairs assignment; for example, PEM had a 0% probability of being assigned as PUA, whereas PUA had a 60% probability of being assigned as PEM.

**Table 3:** Percentages of pairs posterior assignment probability of each group.

Assign groups	FOM	APG	PRA	PUA	PEM
FOM	/	46,10	1,45	0,00	75,45
APG	32,99	/	11,59	0,71	23,95
PRA	1,55	34,20	/	99,29	0,00
PUA	0,00	2,90	71,43	/	0,60
PEM	65,46	15,99	0,00	0,00	/

Forage mixtures (FOM); First alfalfa cutting with the prevalence of graminaceous >50% (APG); Prevailing alfalfa >50% (PRA); Purity alfalfa >95% (PUA); Permanent meadows (PEM).

## 4. Discussion

The nutritive value of forages is determined by genetics (grass species), phenological state, and environmental factors such as soil, weather conditions, grazing, and cutting management. Hay classification is a useful practice to properly store homogeneous production bales to be included in the Total Mixed Ratio (TMR) of lactating cows: classification is essential for validating the hay quality results from NIRS analysis, and it must be performed before the addition in the mixer wagon or distribution in the feeding lane. The usefulness of the NIRS application directly on the mixer wagon for control and management of the characteristics of the raw materials has been documented in a recent review by Evangelista et al. in 2021 [37] even if some limits as calibration and interpretation procedures specific for the hay of the area, high investment costs for the equipment and low level of technologization of farmers could be considered. However, if hay characteristics are known, evaluators or breeders can quickly classify hay and other directly or indirectly related quality factors while laboratory analysis become complementary. Up to now, multivariate analysis has been used in forage evaluation and classification: some authors presented multivariate analysis to calculate the energy forage index [25], to calculate the variation in yield and nutritive value [38], to estimate protein content and in vitro dry matter digestibility [39], and to investigate the influence of quality parameters on the methanogenic potential of different forages [40].

From the presented study emerged that it is possible to identify 2 groups of hay with two different multivariate statistical approaches: the first group of matrices with alfalfa (PUA and PRA) and the second group (FOM, PEM, and APG) composed of Gramineae species. The correct classification of these 2 groups, which are generally included in the rations of lactating cows, was an interesting result in practical use. In the Parmigiano Reggiano system, where hay is the base of TMR in feeding dairy cows, the main problem is optimizing the alfalfa grass mix. For this reason, in recent years, there has been a progressive specialization of forage crops: alfalfa (PUA and PRA) is increasingly cultivated in pure stands to obtain high-quality protein forage, while grasses (FOM, PEM, and APG), which remain essential for providing the necessary fiber in the ration, come from permanent meadows or pure stands of Italian ryegrass or winter wheat. Furthermore, in the last years in Italy, alfalfa is becoming one of the most spread cultivars due to its adaptation capacity to different conditions, the nutritional value for ruminants, and its potential productivity [41].

The chemical analysis of our samples was generally similar to those summarized by the Dairy NRC (2001), indicating that a representative range of hay was included in this study. Excluding the PUA group, protein level (CP) seemed, on average, lower than those reported by [42], who evaluated samples of meadows and grasslands located in north-central Apennine (Italy), while ADL seemed to be similar. The content of CP of our sample was slightly higher for all types of hays and especially for prevalent alfalfa hays (PUA and PRA) with respect to those reported by Zicarelli et al. in 2022 [43], which considered different altitude areas of Campania Region. The different protein content of hays could be affected by various factors, firstly the different climate conditions and endogenous characteristics between the Regions. However, chemical hay parameters are strongly affected by the phenological state, the number of cuts, and the age of the meadows as well as the human management [41]. The first cut of alfalfa (PRA) is usually a mixture of native grasses with a limited contribution of alfalfa. Following grasses regrow slower than alfalfa, reducing their proportion in subsequent cuttings. Hays are highly variable in composition, digestibility, and intake potential, and understanding the factors that affect this fluctuation is important for making skillful evaluations. However, the Italian hays are often of low quality and reduced nutrient value due to the influences of climatic conditions [6] or the management of their cuts. As reported in a previous study [6], hay groups characterized by alfalfa (PRA and PUA) showed high protein contents and a lower level of aNDFom with lower digestibility after 24h of in situ rumen incubations. Grasses (FOM, APG, and PEM) showed high aNDFom digestibility and uNDF lower content, and it is possible to note the correlations between the digestibility of forages with NDF and its lignification [44]. According to [45], mineral contents of forages were low both for Mg and Ca with a negative correlation with dNDF and aNDFom parameters.

### 4.1 PCA and DAPC analysis

Two components were retained from PCA, explaining 54.4% of the total variability. The PCA plot showed the physiological change in the botanical composition of PUA towards PRA and then APG, depending on the agrotechnical management, cutting, climate, edaphic, and biological conditions under which the PUA is developing. In conjunction with DAPC analysis, PCA resulted in a valuable differentiation and posterior assignment among five hay types. Previous work [46] had only quantified the proportion of perennial ryegrass cultivars in intra-species mixtures using discriminant analysis; Gallo et al. [25] used principal component analysis to classify forages and predicted their calculated energy content. A reliable approach to evaluating hay quality is a combination of visual combined inspection with NIRS analysis. However, more studies are desirable to validate the reliability of sensory analysis with NIRS.

Twelve principal components, explaining ~60% of the total variability of original data, were used in the discriminant analysis of principal components. The overall assignment success rate to the group of origin was 66%. Alfalfa is a perennial legume with unique anatomy comprising relatively distinct protein-containing leaves and fibrous stems [32]; for these specific characteristics, the assignment success for PUA was the highest (84%) among the five groups studied, followed by FOM (79%), PRA (69%), APG (37%), and finally PEM with an overall assignment success rate of 27% to the group of origin. In the posterior assignment, APG resulted in a very heterogeneous group, which aligns with the characteristic of commercially available forage mixtures. FOM and PEM overlapping was clear, underlining the difficulty distinguishing samples from these two groups extremely admixed. The percentage values of the pairs were not interchangeable, which is expected due to the lack of the pair’s reciprocity in this type of approach. The two scenarios realized with the DAPC analysis have shown interesting results regarding the different hays’ training, validation, and posterior assignment probability.

## 5. Conclusion

Discriminant analysis can be successfully used to differentiate hay types and could also be used to assess factors related to hay quality in addition to NIRS or chemical analysis. The newly developed method provided a complementary way to differentiate hay types and classify hay samples, obtaining a good percentage of successful assignments, especially for hay where alfalfa prevails.

This type of classification is strongly affected by the number of incoming information but has the advantage that the system can be continuously fed once new acquisitions are made available. The further steps to be taken are, therefore, to increase the amount of information available in order to obtain increasingly accurate estimates and to make the classification system available in an automated way in the agricultural equipment used for haymaking. The changed climatic context has lengthened the haymaking period in autumn, making further investigations into the types of hay and their quality necessary. This additional contribution on the knowledge of the nutritional characteristics of hays will allow a better inclusion of the different types in diets.

## Supporting information

S1 Fig Monthly mean minimum (Tmin) and maximum (Tmax) air temperatures and total rainfall recorded during the six growing seasons at a sampling site representative of the study area. (TIF)
