# Supplementary figures and images for: Discriminant analysis as a tool to classify farm hay in dairy farms
Source: PLoS One. 2023 Nov 28;18(11):e0294468. doi: 10.1371/journal.pone.0294468 (PMC10684012; doi:10.1371/journal.pone.0294468)

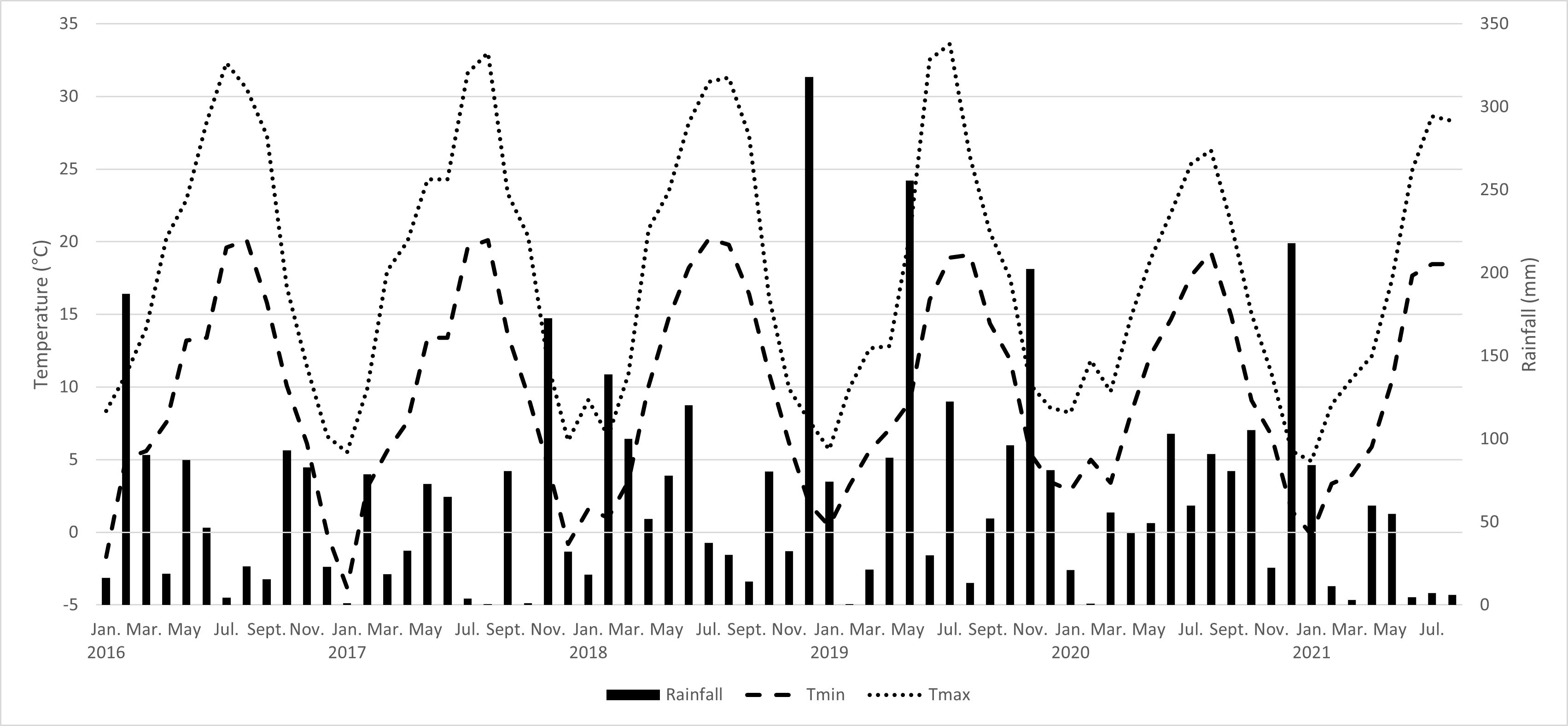

Supplement: S1 Fig — (TIF) [file pone.0294468.s001.tif]
